# Psychological characteristics of the relationship between mental health and hardiness of Ukrainians during the war

**DOI:** 10.3389/fpsyg.2023.1282326

**Published:** 2023-11-10

**Authors:** Viktoriia Predko, Manuel Schabus, Ivan Danyliuk

**Affiliations:** ^1^Department of General Psychology, Faculty of Psychology, Taras Shevchenko National University of Kyiv, Kyiv, Ukraine; ^2^Department of Psychology, University of Salzburg, Salzburg, Austria; ^3^Department of Experimental and Applied Psychology, Faculty of Psychology, Taras Shevchenko National University of Kyiv, Kyiv, Ukraine

**Keywords:** mental health, hardiness, Ukrainians, war, resilience

## Abstract

**Objectives:**

The aim of our survey is to identify psychological features for the relationship between mental health and hardiness of Ukrainians during the war.

**Methods:**

The study involved 608 Ukrainians. We aimed to identify the relationship between mental health and hardiness and determine the differences in the peculiarities of mental health of people with different levels of hardiness. Also we looked for predictors for hardiness.

**Results:**

Subjective hardiness was found to be related to specific manifestation of mental health in the Ukrainian population. Strong correlations were revealed for hardiness with adaptation (ρ = 0.818), emotional comfort (ρ = 0.786), internality (ρ = 0.672), self-perception (ρ = 0.656,), escapism (ρ = −0.632) and mental health (ρ = 0.629). A prognostic model based on linear regression analysis identified the main predictors of personal hardiness and confirmed correlational analysis. Mental health (0.341), emotional comfort (ρ = 0.786), and escapism (−0.576) were found to be good predictors. Altogether 40.1% of Ukrainians scored low, 54.6% medium, and 5.3% high on individual hardiness.

**Conclusion:**

The study found that about every 4th Ukrainian demonstrates a low level of personal hardiness, which is accompanied by emotional discomfort and lack of internal locus of control, making them more susceptible to stress and illness. Additionally, they tend to distance themselves which significantly exacerbates the situation. It has been found that Ukrainians with low levels of personal hardiness exhibit escapism (with diversion of the mind to imaginative activity), a destructive defense mechanism that not only prevents effective problem solving but also has long-term negative consequences for their overall health. Consequently, especially people with low hardiness should receive specific support to stabilize their mental wellbeing and health overall.

## 1. Introduction

Mental health of Ukrainians is becoming a central issue today, as many people who have survived the war develop serious mental and social disorders. War, which destroys normal life, is a major factor of psychological vulnerability (Mollica et al., 2004). In psychological literature, post-traumatic stress disorder is often linked to concept of “war neurosis” (McFarlane, 1995). Moreover, studies show that the secondary effects of war are the main predictors of psychological problems. War injuries, paraplegia, or amputation increase the likelihood of later psychological dysfunction in the individual (Bracken et al., 1995). War stress is the most powerful pathogenic factor involved in the emergence, detection and exacerbation of somatic, mental and behavioral disorders (Figley et al., 2013). The literature has described people’s reactions to adversity based on the life-changing model (Dohrenwend and Dohrenwend, 1974), which views any change as stressful and potentially dangerous. This model suggests that people will react to war with regression and exacerbation of symptoms, which reinforce pathological reactions, such as distorted perception of reality, negative emotional reactions, sleep and eating disorders. The most common and visible psychological effects of war are acute and post-traumatic stress disorder (PTSD; Solomon et al., 1997). It has been documented that civilians exposed to the bombing develop post-traumatic stress disorder, anxiety, depression, substance abuse, and functional impairment. Exposure to the bombing also indirectly affects the effects of the disaster through changes in worldview and one’s own abilities, namely the bombing can modify the personality structure – self-configuration – and ultimately lead to changes in systems of meaning and ideology (e.g., beliefs or values about the world and people in general), emotion regulation systems, and patterns of hardiness and coping. Also, refugees are at excessive risk for psychiatric morbidity due to forced migration and resettlement in unfamiliar environments. One in 10 refugees in Western countries have PTSD, about one in 20 have major depression, and about one in 25 have generalized anxiety disorder. Everyone’s emotional distress on a dyadic level will affect relationships and with others, impaired control of anxious emotions, leads to inadequate coping and negative feelings, difficulty in regulating internal experiences, persistence of negative working patterns of self and others, and ultimately, long-term PTSD (Florian et al., 1995). Thus, the negative impact of the traumatic events of the war has significantly affected the mental health and level of functioning of the Ukrainian population. Psychological consequences are multifaceted and their manifestations are not limited to psychiatric symptoms and syndromes. They are also seen in profound changes in the long-established cognitive pattern about meaning, value, and goodness of self and the world (Janoff-Bulman, 1989).

In research, it has been found that victims of combat stress reactions exhibit low self-esteem and reduced faith in people’s benevolence. After traumatic events, they perceive the world as very dangerous and threatening, and they do not see themselves as worthy and protected (Janoff-Bulman, 1989). A decrease in the subject’s self-esteem, lack of faith in one’s good luck, benevolence of the world and other people leads to deterioration and decline in mental health (Kaler, 2009); moreover, hostility and blaming others is directly related to depressive symptoms, obsessive-compulsive behavior, etc. (Lahav et al., 2016). It is worth noting that humans are the only species that can survive the most unfavorable experiences, are able to adapt to changing circumstances focus on their own unique experience, analyze it and rethink it, and thus demonstrate hardiness. The war entails a wide range of hardships, which contains many negative factors and has a destructive effect on the psychological development of the individual, but it is the reassessment of the experience that will help not only to overcome the hardships but also to show inner potential.

There is no single standard by which mental health can be measured, diagnosed and examined. Current approaches are based on the concept of mental health as a manifestation of a high level of physical, mental and social well-being (Vera-Villarroel et al., 2014). Moreover, the World Health Organization for a long time has not focused exclusively on the absence of disease, but rather on the need to include subjective psychological parameters that support health and well-being (World Health Organization, 2005). The notions of mental health as a positive self-actualizing force and of personality as a unified and coherent organization of experience have become increasingly evident in recent publications. Accordingly, the main psychological aspects of mental health are healthy motivation, self-esteem, locus of control, self-perception and self-efficacy (Meade et al., 2023).

People have a set of stable assumptions about the world that help predict events, interpret information, guide perceptions, and make decisions (Janoff-Bulman, 1989). These assumptions are the basis of our good will because they give us the feeling of a certain kind of irreducibility. Janoff-Bulman identified eight basic worldview assumptions, which can be divided into three main categories: the goodness of the world, the importance of the world, and the goodness of one’s own life. According to Janoff-Bulman, people begin life with a positive set of assumptions about the world, which usually support a sense of invulnerability and optimism in everyday life (Janoff-Bulman, 1989). However, in the case of a traumatic event, they break down, which is a major aspect of post-traumatic stress, a change in the perception of the self and the world. It is the change in these most fundamental schemas, deeply rooted in our conceptual system, that is at stake in the case of traumatic life events (Janoff-Bulman, 1989). The tension between a person’s conceptions of the world and newly received information about traumatic events persists, which can also lead to psychopathology. In addition, the destruction of world assumptions affects various subsystems of the individual-personal structure, changing the individual’s physical, behavioral, emotional, social and cognitive functioning. Among the most significant cognitions associated with the severity of PTSD symptoms are beliefs about the hostility and danger of the outside world and beliefs about self as weak and incompetent. That is, the individual directly confronts the terror of the world around him or her, as well as his or her own vulnerability and helplessness; accordingly, the pre-existing confidence in one’s own safety and invulnerability turns into an illusion, plunging the individual into a disintegrating state. Consequently, in most cases, survivors of stress or trauma suffer a destruction of their beliefs and subsequently maintain negative perceptions of self and the world, characterizing the world as less welcoming, which in turn further increases their vulnerability to posttraumatic stress disorder (PTSD; Janoff-Bulman, 1989).

Moreover, cognitions are one of the important factors in how people cope with traumatic events. World assumptions (WAS) are not only key cognitive schemas through which people interpret the world, themselves, and others, but also key indicators of mental health (Janoff-Bulman, 1989). In general, the relevant cognitive schemas are more closely related to general indicators of mental health and functioning than to specific reactions characteristic of trauma. It is important to note that cognitive schemas are negatively altered in response to distress in general, not just as a result of traumatic exposure. The process of coping with trauma, according to Janoff-Bulman, consists in the restoration of basic beliefs: if successful, they become qualitatively different from what they were before the trauma, but the restoration does not occur completely, but only to a certain level, freeing the person from the illusion of their own invulnerability. The picture of the world of an individual who has experienced a psychological trauma and successfully coped with it acquires a qualitatively new level: “The world is benevolent and fair to me. I have the right to choose. But this is not always the case” (Janoff-Bulman, 1989).

Many of the negative effects of war on physical and mental health are also related to chronic stress. The physical effects of chronic stress caused by military operations can perpetuate the state of allostatic stress in the body and accelerate disease processes. Chronic stress has a negative impact on mental health, leading to depression, anxiety, and a general decline in quality of life. People differ in their ability to cope with stressors. Whether a person capitulates to the negative effects of stress depends in part on his or her ability to apply physical or psychological coping strategies. According to the COR theory, people need resources to overcome stress and maintain mental health. Such resources include self-efficacy, empathy, social responsibility, autonomy, active participation in problem solving, etc. (Hobfoll et al., 1992). The coping model is based on behavioral rather than emotional coping strategies. It is known that problem-oriented coping is the most effective strategy. It is superior to avoidance, which is not about directly solving the problem but about taking preparatory measures to solve the problem, passivity, and avoidance of decision-making. It is worth noting that the model of coping conceptualizes stress in the context of interpersonal relationships, since most stress is not only an individual but also a common, shared problem. In this theory, human actions are considered in relation to their social environment, i.e., a person preserves not only his or her integrity but also the integrity of society. That is, prosocial strategies are important factors in successful overcoming of stress, integrating both individual and collective coping mechanisms (Hobfoll et al., 1992).

Mental health is a complex construct that encompasses both affect and psychological functioning from two different perspectives: the hedonic perspective, which focuses on the subjective experience of happiness and life satisfaction, and the eudemonic perspective, which focuses on psychological functioning and self-realization (Hobfoll et al., 1992). A favorable level of mental health is determined by mental well-being, effective functioning of both an individual and a community. It is a state that allows people to realize their abilities, cope with life stresses, solve problems productively, and contribute to their society. Poor mental health limits the manifestation of both eudemonic and hedonic well-being of the individual and society as a whole (Hobfoll et al., 1992).

In general, people have the hardiness to survive even the most adverse events, and most war survivors who have experienced traumatic events are able to adapt to changed circumstances and integrate extreme experiences into their cognitive structure. However, this process of adaptation is not always successful, as a significant share of the population suffers from trauma, which manifests in mental health complaints, including post-traumatic stress disorder (PTSD) and/or depression (Kilpatrick et al., 2013). Thus, research shows that the “level of exposure” to stressors is a stronger predictor of negative mental health outcomes than direct military exposure (Miller and Rasmussen, 2010). While stress can indeed have a physiological effect on individuals, it seems that certain personality traits, such hardiness as can help alleviate its impact (Kobasa, 1979). In the early 80s of the 20th century, American psychologists Suzanne Kobasa and Salvatore Maddi coined the term “hardiness” to define the psychological, worldview beliefs of a person that prevent the negative effects of stress, allow a person to remain active and confident in a favorable resolution of a difficult situation, provide hope and motivate to solve the problem, ensure transformational overcoming of stress and difficult life situations. In particular, they act as a psychological buffer against stress sensitivity (Maddi, 2004).

According to Salvatore Maddi’s theory, hardiness has three components: engagement (activity, initiative, interest in the world around us), control (the ability to recognize, realize, control and adjust one’s emotions in time) and challenge (the ability to learn from unpleasant events and see a positive meaning in them; Maddi, 2004). It is manifested as the ability to gain positive experience from difficult life events, as the ability to acquire new skills and abilities, to transform one’s worldview, reassess values, realize a new meaning, and see what will give one the strength to live on. Personal hardiness is linked to mental health, which manifests itself in the physical, mental and social aspects of life (Farivar et al., 2007). S. Maddi emphasizes the importance of all three components for maintaining health and optimal performance and activity in stressful conditions (Maddi, 2004). Thus, the psychological mechanism by which a person transforms difficult life situations into more favorable experiences contains a combination of survival strategies that are related to the components of inclusion and control. These strategies enable a person to stay in a difficult situation through his or her commitment to their values and goals and, at the same time, to manage the situation and its consequences (Florian et al., 1995). The challenge component provides an opportunity to perceive a difficult life situation as a challenge, and to respond well to any changes. The risk component contributes to finding meaning in one’s life and to the desire to gain experience. Challenge orientation is expressed in the belief that change, not stability, is a normal, constructive phenomenon in life. It is true that the expectation of change is an interesting stimulus for personal growth, and not a threat of danger. The challenge reduces the level of perception of the stressfulness of the situation. It leads to attempts to transform oneself, to self-renewal and self-realization, instead of preserving the past pattern of existence. Thus, initially a person finds meaning for himself in a life situation, then he or she forms a goal that corresponds to his or her basic values and, only then, a person becomes capable to evaluate potential advantages of a difficult life situation and perceive it as a leading opportunity for personal development (Taylor, 1983). In particular, A. Maslow noted that the most significant experience in the life of an individual who has achieved self-actualization is a tragedy, under the influence of which he or she was forced to look at life differently, to change his perception of the world (Maslow, 1970). In logotherapy, it is generally accepted that it is suffering gives rise to the best qualities in a person, changes him or her, makes him or her wiser, and provides an opportunity to understand the meaning of life. The connection between the meaning of life, higher values and self-transcendence is most strongly manifested in a difficult life situation, because for the sake of our values we are ready to accept suffering and sorrow, we can endure inevitable suffering and at the same time not break down (Batthyány, 2017).

Hardiness exists on three levels: psychophysiological (optimal reactions to stress), socio-psychological (effective self-regulation) and personal-semantic (meaningfulness and positive outlook; Maddi, 2002). Maddi’s model of hardiness includes not only the construction of these three components, but also five basic psychological mechanisms:

- Stable beliefs – a person’s perception of any events in life as safe.
- Motivation of a person to transform, accompanied by openness to everything new, a person’s readiness to act actively in difficult life situations, using constructive behavioral strategies.
- Increasing the body’s immune response through mental and physical mobilization.
- Increased responsibility and care for one’s own health.
- Search for social and psychological support through the development of communication skills (Maddi, 2002).

Hardiness moderates the impact of stress on health by influencing how individuals perceive and respond to challenging situations. Hardy individuals tend to view novel and disruptive life events as normal aspects of life, which they believe they can handle. When faced with stress, they are inclined to employ proactive problem-solving strategies, while those lacking hardiness tend to resort to avoidance tactics like denial or substance use. Therefore, hardy individuals initially view stressors positively and actively seek solutions to adapt to new circumstances (Bartone et al., 2022). The current research suggests that hardiness serves as a valuable resilience factor, offering a degree of defense against depression among soldiers facing intense combat stress. Importantly, this protective role is primarily attributed to the coping mechanisms employed by these soldiers. Those with lower levels of hardiness tend to rely more on avoidance coping strategies, which can subsequently result in higher levels of depression symptoms and associated difficulties (Bartone and Homish, 2020). Hardiness, may provide a safeguard for individuals with extensive military backgrounds. The study’s findings suggest that individuals with high levels of hardiness may possess a form of resilience that guards them against the impacts of PTSD, even after extended military service (Escolas et al., 2013). Hardiness serves as a defense against the negative consequences of stress, especially when faced with high levels of stress or multiple stressful situations (Bartone, 1999). Thе hardiness contributed to increased stress resiliency, as measured by a lower level of reported mental health complaints (Thomassen et al., 2015).

Thus, the term “hardiness” is an important internal resource, a combination of current attitudes and formed skills, attitudes and beliefs, which provide an opportunity successfully adapt to stressful situations, and ensure that a person overcomes difficult life situations.

## 2. Materials and methods

We contacted Ukrainians in the time frame between 1st of April 2023 to 1st of June 2023.The survey was distributed and administered online. The data for this study were collected using Google Forms with proper instructions and explanations for completion. The participants of the study were university students of all higher education institutions in Ukraine, attendees of public services, and refugee centers: participants were also asked to share the questionnaire among their relatives and friends. We also using individual contacts of authors, University mailing lists and public announcements in the Ukraine. The main inclusion criteria for the participants were age, being a citizen of Ukraine, either living in different territories of Ukraine, being refugees or temporary migrant in other countries. The study did not presume any specific exclusion criteria.

Participants with missing responses have been removed leaving the analyzable sample to 608 Ukrainians. The age range of the sample varies from 18 to 61, with female 87.5 and 12.5% male. The limited number of men taking part in the survey might be related to their involvement in the armed forces leaving them with limited/controlled access to the information online.

For this study the following questionnaires were used:

Rogers-Diamond Test of Personal Adjustment (Rogers, 1961). We used the adaptation into Russian by Osnitsky (2004). It assesses personality adaptation through 101 statements, evaluating overall adjustment, acceptance of others, internality, self-perception, emotional comfort, desire to dominate, and escapism. World Assumptions Scale (WAS): Measures assumptions and beliefs about the world after life-altering events or traumatic experiences (Janoff-Bulman, 1989). The study used Russian adaptation made by Padun and Kotelnikova (2008). It consists of 32 items in subscales: benevolence of the world, meaningfulness, worthiness of the self. Mental Health Continuum Short Form (MHC-SF). The current study used the Russian adaptation made by Osin and Leontiev (2020). It has 14 items measuring emotional, psychological, and social well-being when combined, eudemonic well-being. SF-12 Health Survey. Questionnaire in Russian adaptation by Feshchenko et al. (2002). It is a 12-item assessment of generic health and health-related quality of life from the client/patient’s point of view. The Strategic Approach to Coping Scale (SACS), Russian adaptation by Vodopjanovaja and Starchenkovaja (2002). It assesses coping strategies via 54 statements in 9 behavior models to handle stressors (1) assertive actions; (2) entering into social contact; (3) seeking social support; (4) careful actions; (5) impulsive actions; (6) avoidance; (7) manipulative (indirect) actions; (8) antisocial actions; (9) aggressive actions. Russian language adaptation of Salvatore Maddi’s Hardiness Survey by Osin and Rasskazova (2013a). The test is designed to measure major components of hardiness (control, communication, and challenge) and overall hardiness. The questionnaires were administered using online google forms and lasted on average 60 min for all surveys together. The results of the statistical analysis of hardiness indicators were compared with the normative data (Average value 50.79; Low values 39 and below; High values 62 and above; Osin and Rasskazova, 2013b), thanks to which it was possible to distinguish groups of individuals with different levels of hardiness, namely the group of individuals with low hardiness 40.1%, medium 54.6%, and high 5.3%. Given multiple comparisons (148 correlations) we corrected using Bonferroni correction and only consider results with values of *p* < 0.001 as significant in the following.

## 3. Results

Statistical analysis was performed using SPSS (version 29). Spearman’s correlation analysis was used to identify the relationship between mental health and hardiness. One-factor analyses of variance (ANOVA) were used to determine the differences in the peculiarities of mental health of people with different levels of hardiness. Regression analyses were used to determine the predictors of hardiness.

At the first stage of the study, we conducted correlation analysis to identify the link between personal hardiness and the peculiarities of mental health during the war. The results are presented in Table 1.

**Table 1:** Correlations between hardiness and mental health manifestations.

Variables	Hardiness
Adaptation	0.818*
Emotional comfort	0.786*
Internality	0.672*
Mental health	0.629*
Acceptance of others	0.568*
Emotional well-being	0.206*
Benevolence of world	0.154*
Social well-being	0.145*
Escapism	−0.632*
Self-perception	0.656*
The desire to dominate	0.473*

Note: Bold text indicates a significant Spearman correlation after Bonferroni correction. *p < 0.001.

We found strong positive correlation between personal hardiness and: emotional comfort (ρ = 0.786, *p* < 0.001), internality (ρ = 0.672, *p* < 0.001), self-perception (ρ = 0.656, *p* < 0.001), mental health (ρ = 0.629, *p* < 0.001), acceptance of others (ρ = 0.568, *p* < 0.001), the desire for dominance (ρ = 0.473, *p* < 0.001); emotional well-being (ρ = 0.206, *p* < 0.001); the general attitude toward the benevolence of the world (ρ = 0.154, *p* < 0.001), social well-being (ρ = 0.145, *p* < 0.001), adaptation (ρ = 0.818, *p* < 0.001). Also, it was revealed a negative correlation between escapism and hardiness (ρ = −0.632, *p* < 0.001).

**Figure 1:**
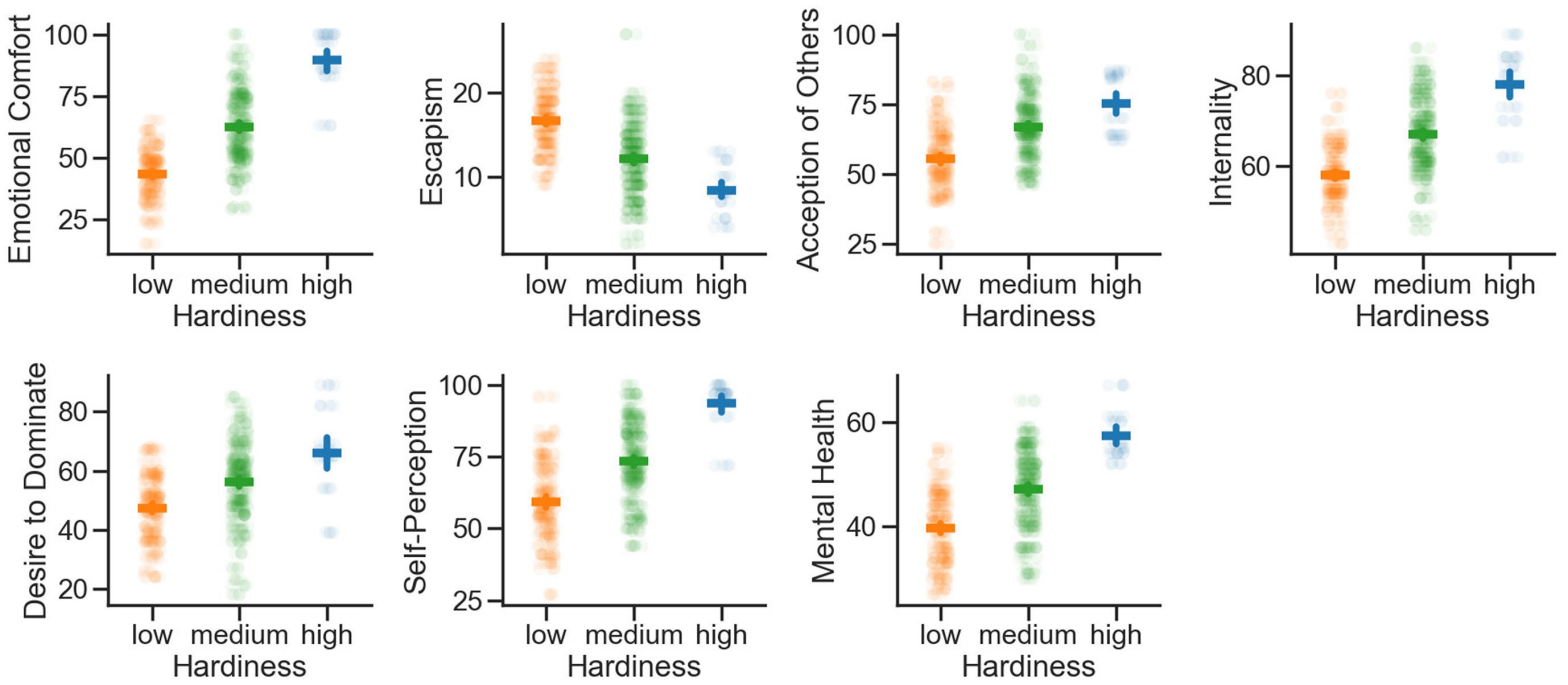
Differences in the peculiarities of mental health of people with different levels of hardiness. Note that people with high hardiness always score higher on acceptance of others, the desire to dominate, internalization, escapism, mental health and especially for the variables emotional comfort and self-perception. The y-axis of the depicted variables is always having a range from 0 to 100 with the exception of escapism (here the authors Rojers and Daymond consider values >10 as low and >20 as high escapism), desire to dominate (values >20 as low and >80 as high), internality (values >60 as low and >80 as high). The mental health variable is also having a range from 0 to 60, with values >60 being considered a good mental health and values <40 being considered low mental health. The different colors indicate the respective levels of hardiness (low, medium, high). The horizontal colored bars indicate the mean and the vertical colored bars the related 95% confidence interval. Single subjects are presented as dots with a high opacity to better show how the peculiarities of mental health are expressed on the individual.

At the second stage of this study, we conducted a one-factor analysis of variance ANOVA (Schaeffer’s method) to identify differences in the specific manifestations of mental health issues in people with different levels of hardiness (Figure 1).

The ANOVA reveals significant differences in self-perception (individual’s self-awareness and perception of themselves, including their strengths and weaknesses) between the three groups of hardiness [*F* (2,605) = 137.751; *p* < 0.001]. *Post-hoc* tests indicated that specifically, individuals with a high level of hardiness exhibited higher self-perception scores (*M* = 93.6, *SD* = 8.91) compared to those with medium (*M* = 73.4, *SD* = 13.4 *p* < 0.001) and low (*M* = 59.3, *SD* = 13.74, *p* < 0.001) levels of hardiness. Individuals with medium hardiness compared to those with low hardiness had greater self-perception (*p* < 0.001).

Furthermore, significant group differences are observed for acceptance of others [*F* (2,605) = 89.003; *p* < 0.001]. Analyses indicated that individuals with low hardiness displayed the lowest acceptance of others (*M* = 55.5, *SD* = 11.71) compared to the medium (*M* = 66.9, *SD* = 11.4, *p* < 0.001) and high (*M* = 75.2, *SD* = 10.66, *p* < 0.001) hardiness groups. Individuals with medium hardiness, when compared to those with high hardiness, exhibit lower levels of acceptance of others (*p* < 0.001).

In terms of emotional comfort, a significant difference is identified across the three groups of individuals [F (2,605) = 271.100; *p* < 0.001]. Specifically, individuals with high levels of hardiness reported the highest еmotional comfort scores (*M* = 89.3, *SD* = 12.21) compared to individuals with low hardiness who reported the lowest scores (*M* = 43.2, *SD* = 9.9, *p* < 0.001) and individuals with medium hardiness who fell in between (*M* = 62.4, *SD* = 14.65, *p* < 0.001). Furthermore, individuals with medium hardiness, compared to those with low hardiness, exhibited significantly higher levels of emotional comfort (*p* < 0.001).

Moreover, significant group differences are observed regarding the desire to dominate [*F* (2,605) = 51,340; *p* < 0.001]. Individuals with high levels of hardiness showed a higher desire to dominate (*M* = 66.1, SD = 14.69) than individuals with medium (*M* = 56, *SD* = 13.62, *p* < 0.001) hardiness, as well as individuals with low hardiness levels who exhibited the lowest desire to dominate (*M* = 47.3, *SD* = 10.89, *p* < 0.001). Individuals with medium hardiness, in comparison to those with low hardiness, showed a significantly higher desire to dominate (*p* < 0.001).

The pattern continues with internalization (level of subjective сontrol, the degree to which an individual believes they have control over their own life), which significantly differs among different levels of hardiness [*F*(2,605) = 89.003; *p* < 0.001]: individuals in the low hardiness level group recorded the lowest internalization scores (*M* = 58.1, *SD* = 6.39) compared to the medium (*M* = 66.9, *SD* = 8.38, *p* < 0.001) and high (*M* = 78, *SD* = 8.49, *p* < 0.001) hardiness groups. Additionally, individuals with medium hardiness exhibited lower internalization compared to those with high hardiness (*p* < 0.001).

However, escapism (the tendency of individuals to seek escape from reality or unpleasant situations by diverting themselves toward alternative goals or avoiding problems) is also significantly different among individuals with different levels of hardiness [*F* (2,605) = 119.422; *p* < 0.001]. But, individuals with low level of hardiness scored higher (*M* = 16.6, *SD* = 3.55) in comparison to those with medium level of hardiness (*M* = 12.1, *SD* = 4.44, *p* < 0.001) and individuals with high hardiness levels scored the lowest escapism (*M* = 8.3, *SD* = 3). Furthermore, individuals with medium hardiness exhibited a higher escapism score compared to those with high hardiness (*p* < 0.001).

Regarding mental health, significant differences are observed [F (2,605) = 128.698; *p* < 0.001]. People in the low hardiness level group scored the lowest in mental health (*M* = 39.7, *SD* = 6.97) compared to the medium (*M* = 47.0, *SD* = 7.44, *p* < 0.001) and high (*M* = 57.3, *SD* = 4.7, *p* < 0.001) hardiness groups. Additionally, individuals with medium hardiness, when compared to those with high hardiness, exhibited a lower mental health score (*p* < 0.001).

Finally, we computed a prognostic model using linear regression. Multiple regression analysis was used to identify the main significant predictors of personal hardiness. The results of the regression indicated the predictors explained 75.8% of the variance. The results are presented in Table 2.

**Table 2:** Variable results of linear regression showing predictors of hardiness.

	Unstandardized coefficients		Standardized coefficients	*t*	*p*	Collinearity statistics	
	*b*	*SE*	β			*Tolerance*	*VIF*
Mental health	0.341	0.041	0.219	8.286	<0.001	0.581	1.722
Self-perception	0.108	0.026	0.131	4.086	<0.001	0.397	2.521
Emotional comfort	0.218	0.028	0.290	7.735	<0.001	0.287	3.479
Internality	0.200	0.041	0.141	4.871	<0.001	0.485	2.060
Escapism	−0.576	0.073	−0.207	−7.84	<0.001	0.583	1.716
Emotional well-being	0.306	0.089	0.076	3.433	<0.001	0.833	1.201
Meaningfulness of the world	0.097	0.187	0.018	0.520	0.603	0.337	2.969
The desire to dominate	−0.006	0.024	−0.006	−0.250	0.803	0.643	1.554
Avoidance	−0.127	0.072	−0.038	−1.76	0.077	0.865	1.156
Controllability	0.160	0.135	0.041	1.183	0.237	0.337	2.970
Acceptance of others	0.104	0.028	0.103	3.668	<0.001	0.518	1.929

The fitted regression model was: Hardiness = −7.465 + 0.341 * (mental health) + 0.306 *(emotional well-being) + 0.218 * (emotional comfort) + 0.200 * (internality) + 0.160 * (controllability) + 0.108 * (self-perception) + 0.104 * (acceptance of others) + 0.097 * (meaningfulness of the world) + (−0.06) * (the desire to dominate) −0.127 * (avoidance) + (−0.576) *(escapism). The overall regression was statistically significant [*R^2^* = 0.758, *F*(11, 596) = 170.065, *p* < 0.001]. It was found that higher mental health (*β* = 0.219, *p* < 0.001), lower escapism tendencies (*β* = −0.207, *p* < 0.001), and higher emotional comfort (*β* = 0.290, *p* < 0.001) were the strongest predictors for hardiness.

## 4. Disсussion

The conflict and war between Russia and Ukraine has totally destroyed the established way of life, annihilated peace and concord. It causes constant stress and frustration, which, in turn, activate neurobiological reactions and psychological defense mechanisms. These protective mechanisms act as barriers to stabilization of the general condition of the population against the backdrop of crisis phenomena, and they could impede effective life of every member of the Ukrainian society. At the same time, the war forces people to react quickly and survive, to find ways to minimize anxiety and to form a personal sense for a successful overcoming of difficulties. In this, they are helped by hardiness – an internal resource of a person, which has a close relationship with mental health, thanks to which it allows individuals to rethink their life beliefs, find meaning in all situations, being able to manifest hardiness even in war conditions. It should be noted that mental health is a complex and many-faceted notion, it encompasses general satisfaction with life and personal wellbeing in different walks of life. It includes physical, psychological, emotional, social and spiritual well-being.

During our study, it was found that the manifestation of a high level of personal hardiness is accompanied by adaptation, the ability to effectively adapt even under unfavorable circumstances. That is, personal hardiness prevents an individual from descending into a victim mentality and assists in coping even the most complex life situations. It contributes to emotional comfort and well-being, manifestation of internal locus of control, and psychological readiness to act in a stressful situation. Conversely, a low level of personal hardiness corresponds to a negative psycho-emotional state, internal discomfort, a feeling of powerlessness and hopelessness, and inability to overcome difficulties. In particular, the relationship between hardiness and internality, hardiness and the desire to dominate can be found in scientists’ research, who studied the peculiarities of overcoming difficulties through traumatic memories (Shamsaei et al., 2017). The researchers found that the memories of respondents with a low level of hardiness were dominated by events in which the respondent felt that he or she was unable to influence the situation. Those with a high level of hardiness were dominated by memories in which they successfully overcame difficulties and accepted their own responsibility for the outcome (Shamsaei et al., 2017). Psychological hardiness appears to be a promising factor in enhancing soldiers’ adaptability development. Nevertheless, adaptability always pertains to effective change or adjustment in response to shifting conditions, and there is also evidence that high-hardy soldiers not only adapt better during operational deployments but also adjust more favorably in the months following their return from deployments. All three hardiness facets contribute to soldiers’ adaptive performance. Studies have shown, for instance, that hardiness increases soldiers’ sense of self-efficacy, which, in turn, can lead to more positive and healthy behaviors. Similarly, high-hardy, high-control soldiers will find ways to adapt or adjust the rules to suit a changing situation (Bartone et al., 2013).

It is established that a low level of personal hardiness is accompanied by a tendency to evade problems and reality, rather than addressing them, passivity, and manifestation of destructive, infantile behavioral patterns. Such a tendency serves as a form of psychological defense mechanism, which not only pseudo-minimizes anxiety but also has long-term negative consequences.

Our findings indicate a close interconnection between personal hardiness and good mental health. High level of hardiness prevents heart rate acceleration and increased blood pressure during a stressful situation, respectively, the structural component “involvement” increases the ability of the individual to make the right choice when making a decision, the component “control” reduces internal anxiety, and the structural component “challenge” promotes reassessment of events and stimulates the use of constructive coping strategies (Florian et al., 1995). In Liu’s study, which focused on the relationship between a person’s hardiness and their subjective perception of mental health, it was found that those respondents who showed a higher level of hardiness felt healthy both physically and mentally (Liu et al., 2014). Hardiness curbs the impact of stress on physical and mental health. In addition, it is inversely proportional to depression and anxiety, and it is positively correlated with a person’s psychological well-being (Waters and Cummings, 2000).

Furthermore, we found a strong positive correlation between general attitude toward the benevolence of the world and personal hardiness. The belief that the world is just and secure enables the individual to give meaning to stressors, and to perceive difficulties as a certain sequence that has meaning. Consequently, there is a desire to invest energy and determination in their solution. Conversely, the perception of a threatening, hostile world causes uncertainty, lack of energy, impatience, anxiety, pessimism, and lack of enthusiasm. According to this, it was revealed the relationship between personal hardiness and social well-being, as well as acceptance of others. That is, personal hardiness is closely interconnected with the social well-being of society, and the social well-being of society depends on the readiness of each individual to demonstrate personal hardiness. The social crisis caused by the war lead to changes that make the normal functioning of society impossible, transforms the inner world of each Ukrainian, and reduces personal hardiness. Nevertheless, the ability of the individual to survive this crisis leads to the emergence of new experiences and meaning formation, acts as a certain counteraction to tension and stress, and prevents passive regression.

The second phase of the study revealed significant differences in the expression of mental health based on an individual’s level of hardiness. Individuals with high levels of hardiness have a number of advantages over people with low and medium levels of hardiness. They manifest notably higher self-perception, which gives them confidence in their ability to overcome difficulties. This contributes to robust mental well-being and fosters a constructive approach to challenges. Emotional comfort also displays significant difference: individuals possessing heightened hardiness levels report considerably greater emotional comfort than their counterparts with medium and low hardiness levels. The higher a person’s level of hardiness, the more pronounced is the tendency to dominance and the less pronounced is the tendency to escapism. This pattern is confirmed by the presence of an internal locus of control in individuals with high levels of hardiness. Moreover, the parameter of “acceptance of others” exhibits qualitative differences. Individuals with greater hardiness levels display significantly higher levels of acceptance of others compared to those with lower and medium hardiness levels, which indicates that they tend to show understanding and support, that is an important factor in solving a common problem. People with high hardiness perform significantly better on a measure of overall mental health. According to S. Maddi, hardiness forms a tendency to perceive potentially difficult life situations as less threatening and dangerous. It is a holistic personality characteristic, a general indicator of a person’s mental health, a kind of stress buffer (Maddi, 2004). Individuals with high level of hardiness are less likely to be ill, because high hardiness reduces negative influence of stress on one’s body, ensures ability to adapt during stress, protects from nervous breakdown, and has positive influence on effective problem-solving. In particular, it affects transformative overcoming and posttraumatic growth. Conversely, hardiness is negatively correlated with the regressive problem-solving and overcoming of difficulties by denial and by motivation for failure evasion (Nowack, 1989).

At the same time, a significant number of people characterized by low levels of hardiness indicates that a substantial portion of the Ukrainian population show disbelief in their own strength, demonstrate emotional discomfort and inner tension, which makes them more vulnerable. They have strongly pronounced escapism, tendency to distance themselves from reality, and use destructive coping strategies. These behaviors, in turn, carry potential adverse implications for psychological well-being. Additionally, individuals with low hardiness exhibit a weak internal locus of control, demonstrating a reliance on external circumstances and an inclination to evade assuming responsibility for resolving problematic situations. Simultaneously, they exhibit a tendency to assign blame and criticize others, further jeopardizing their overall well-being. According to the COΡ theory, an individual’s coping with stress is carried out in connection with the social environment, as a person preserves not only his or her integrity but also the integrity of society (Hobfoll et al., 1992). The success of overcoming difficulties is related to the social aspect, interpersonal relations, and cooperation with others, since most of the problems of war are not only individual but also a common problem. Prosocial strategies are an important factor in successful coping with stress, which includes acceptance and tolerance of others. Moreover, Frisby asserts that the expression of hardiness, specifically its structural aspect involving engagement, exemplifies positive conduct and effective social interaction. Conversely, insufficient hardiness primarily leads to discrimination, distrust, intolerance, and discord (Frisby, 2019).

It should be noted that the main predictors of hardiness are mental health, emotional comfort and escapism. Mental health appears to be a kind of reservoir of mental energy and strength that helps people cope with stress and challenges. It is a leading adaptive potential that contributes to personal growth. It allows for flexibility, develops frustration tolerance, and the ability to function optimally even in a situation of uncertainty and also provides the individual with the understanding that life is full of challenges that he or she cannot always control and avoid, but can always remain flexible to changing situations. It manifests itself as optimistic, cognitive and emotional flexibility, the ability to change one’s behavior depending on the situation (Leyro et al., 2010). Notably, positive thinking is associated with strong immunity, increased hardiness, stress resistance, and low anxiety (Cooley et al., 2022). Accordingly, emotional comfort provides the ability to remain calm and confident under the pressure of setbacks, recover quickly from stressful events, and helps regulate negative emotions in a timely manner. Escapism is a destructive coping mechanism that impedes hardiness, as it is based on evasion rather than resolution of the problem. It is accompanied by psychological defense mechanisms, as well as destructive ways to minimize the mental stress caused by the problem situation. Such a strategy impedes the development of reflexivity, meaningfulness of one’s life. In the long run, escapism threatens a disconnection from reality, fostering alienation, loneliness, and a deteriorating psychophysiological state, culminating in increased stress and heightened psychological discomfort.

Turning to the limitations of our study, we need to note that subjects’ self-initiative to partake in the study may skew the sample toward individuals with lower hardiness levels. Those with more mental health issues or lower hardiness might show greater interest in participating compared to their more hardy counterparts. Higher hardiness individuals tend to navigate challenges autonomously and might not feel the need for diagnostics or assistance. The overall sample may be considered insufficient to represent the entire population; however, considering geographical location, age, and other sociodemographic characteristics, this particular sample is capable of showing the main tendencies that are common for entire Ukrainian society, despite women overweighting men. The study is primarily cross-sectional rather than causal in nature. The insufficient number of men participating in the study and the predominantly younger age group narrows the sample. The study surveyed general population and did not take defined specific criterion for differentiating between stress and traumatic experiences. Moreover, hardiness norms may vary due to the specifics of the situation and may not be entirely applicable to this sample.

Nevertheless, indicated limitations represent areas for future research topics that would provide an opportunity to deepen the understanding psychological characteristics of the relationship between mental health and hardiness of Ukrainians during the war.

It can also contribute to the development of effective assistance strategies and support programs aimed at developing and strengthening the hardiness of people during wartime.

It is worth noting that a high level of personal hardiness leads to the formation of a new model of perception that provides meaningfulness, confers value to life, helps to maintain internal balance even in times of war. It is the most important internal resource, which consists not only in avoiding stress, but also in the ability to see positive aspects and meaning even in complex traumatic events, providing existential courage, giving a person’s life value and meaning, and the ability to recover quickly from stressful events.

## 5. Conclusion

The study revealed a close relationship between personal hardiness and mental health, including its main manifestations. Furthermore, the peculiarities of mental health of Ukrainians depend on their level of hardiness. The study found that a significant number of Ukrainians demonstrate a low level of personal hardiness, which is accompanied by emotional discomfort and lack of internal locus of control, making them more susceptible to stress and as a consequence illness. Moreover, it was found that mental health and personal hardiness mutually reinforce each other. Notably, emotional comfort emerges as the primary predictor of hardiness. It is worth noting that humans are the only species that can focus on their own unique experience, analyze it and rethink it, and thus demonstrate hardiness. Given our results we suggest that more specific programs are needed in order to improve psychological wellbeing and mental health in the Ukrainian population and especially in those with low levels of personal hardiness.

## Data availability statement

The datasets presented in this study can be found in online repositories. The names of the repository/repositories and accession number(s) can be found at: https://osf.io/gt9j2/.

## Ethics statement

The studies involving humans were approved by Ethics Committee of the University of Salzburg (EK-GZ 26/2023). The studies were conducted in accordance with the local legislation and institutional requirements. The participants provided their written informed consent to participate in this study.

## Author contributions

VP: Data curation, Investigation, Methodology, Project administration, Resources, Supervision, Validation, Visualization, Conceptualization, Formal analysis, Software, Writing – original draft. MS: Data curation, Investigation, Methodology, Project administration, Resources, Supervision, Validation, Visualization, Funding acquisition, Writing – review & editing. ID: Conceptualization, Data curation, Formal analysis, Methodology, Software, Validation, Writing – review & editing.
